# Unraveling the use of vaccine therapy in HER2-positive breast cancer: a comprehensive review of current state and future perspectives

**DOI:** 10.1186/s13046-026-03789-1

**Published:** 2026-07-25

**Authors:** Aldo Caltavituro, Claudia Martinelli, Michela Palleschi, Miriam Pirolo, Vincenza Cantile, Roberto Buonaiuto, Carmine De Angelis, Fabio Puglisi, Lucia Del Mastro, Grazia Arpino, Mario Giuliano, Michelino De Laurentiis

**Affiliations:** 1 Oncology Unit, Department of Clinical Medicine and Surgery, University of Naples Federico II, Naples, Italy; 2 Clinical and Translational Oncology, Scuola Superiore Meridionale, Naples, Italy; 3 Department of Breast and Thoracic Oncology, Istituto Nazionale Tumori IRCCS “Fondazione G. Pascale”, Naples, Italy; 4 Medical Oncology, Breast & GYN Unit, IRCCS Istituto Romagnolo Per Lo Studio Dei Tumori (IRST) “Dino Amadori”, Meldola, 47014 Italy; 5 Department of Medicine (DMED), University of Udine, Udine, 33100 Italy; 6 Department of Medical Oncology, Centro Di Riferimento Oncologico Di Aviano (CRO), IRCCS, Aviano, 33081 Italy; 7 U.O.C. Clinica Di Oncologia Medica, IRCCS Ospedale Policlinico San Martino, Genoa, Italy; 8 Department of Internal Medicine and Medical Sciences (DiMI), School of Medicine, University of Genova, Genoa, Italy; 9 Translational Genomics and Targeted Therapies in Solid Tumors, August Pi I Sunyer Biomedical Research Institute, Barcelona, Spain

**Keywords:** Vaccine, Breast cancer, HER2 +, Antigens

## Abstract

**Background:**

Despite significant advances in early detection and therapeutic strategies, breast cancer (BC) continues to pose a major public health challenge.

The treatment of HER2-positive (HER2 +) BC has evolved substantially over recent years with the advent of monoclonal antibodies (mAbs), small-molecule tyrosine kinase inhibitors (TKIs), and antibody–drug conjugates (ADCs). Clinical progress has been significantly accelerated by a deeper understanding of the immune-regulatory properties of this subtype and its interactions with the tumor microenvironment.

Despite the availability of effective HER2-targeted therapies, approximately one-third of patients develops resistance, highlighting the need for novel and more durable treatment strategies. Indeed, while current treatments have shown promising efficacy by promoting a “passive immune response,” they are associated with the emergence of resistant clones.

Conversely, vaccine-based therapies are designed to elicit a durable “active immune response” by presenting tumor-associated antigens that stimulate the host immune system to recognize and eliminate malignant cells. This approach has the potential to generate long-lasting immunological memory, preventing recurrence, disease progression, and the emergence of immune-evasive tumor variants.

**Main body:**

This review provides a comprehensive overview of cancer vaccines strategies for HER2 + BC with emphasis on their mechanisms, advantages, limitations, and their current developmental status. We explore diverse delivery platforms, including peptide/protein-based, nucleic acid, and cell-based vaccines. The clinical utility of these strategies is assessed across different disease settings: from prevention and interception in pre-invasive lesions, to the (neo)adjuvant treatment of early-stage BC and the management of advanced metastatic disease. While advanced-stage BC often presents an immunosuppressive tumor microenvironment that hinders vaccine efficacy, early-stage disease offers a more favorable immunological milieu for inducing robust and durable T-cell responses. Furthermore, we discuss emerging innovations such as neoantigen discovery, next-generation adjuvants (e.g., TLR and STING agonists), and novel combinatorial approaches with checkpoint inhibitors to overcome immune evasion.

**Conclusion:**

Although most BC vaccines remain under clinical investigation, they represent a promising frontier for achieving disease control and the coming decade is expected to yield pivotal insights into their clinical utility and therapeutic potential.

## Introduction

As in other malignancies, breast cancer (BC) interacts with the immune system through cancer immunoediting, comprising elimination, equilibrium, and escape phases [1]. In elimination, malignant cells are destroyed, but incomplete clearance often leads to equilibrium. Tumor cells may then evade surveillance, entering the escape phase marked by resistance and unchecked progression [1]. Selective pressure favors subclones with reduced immunogenicity, enabling immune escape through various mechanisms [2].

Historically, BC was considered immunologically “cold” due to low mutational burden and limited antigen diversity [3–5]. Yet evidence supports new immunotherapeutic strategies, especially in HER2-positive (HER2 +) BC. In this subtype, high tumor-infiltrating lymphocytes (TILs) and tertiary lymphoid structures (TLSs) correlate with better outcomes [6], underscoring the role of immune activation. HER2, a key player in BC biology with a known role in cancer cell genesis, proliferation and resistance to therapy, serves both as therapeutic target and tumor-associated antigen (TAA), eliciting responses such as antibody-dependent cytotoxicity (ADCC). Conversely, HER2 + cells exploit immune checkpoints, notably PD-1/PD-L1, to avoid cytotoxicity [7], providing rationale for immunotherapy.

Preclinical data suggest synergy between checkpoint inhibitors and HER2-targeted agents, though clinical results have been modest [8–11]. Consequently, interest is shifting toward alternative immunotherapies, including immune cell engagers (ICEs) [12], chimeric antigen receptor T cells (CAR-T) [13], and cancer vaccines (CVs).

## Classification and mechanism of action

CVs are an evolving frontier in BC immunotherapy [14]. They exploit the immune system’s ability to recognize and eliminate malignant cells with adaptive responses that are typically less toxic than conventional therapies [15, 16]. CVs are being developed for prevention [17], interception [18], and therapy [17]. Prevention refers to vaccinated healthy individuals at risk to prevent cancer from developing it. Interception targets premalignant or early lesions (e.g., DCIS), aiming to halt progression to invasive disease. Therapy involves treating established cancer, typically in the early (neo)adjuvant or metastatic setting, to enhance immune recognition of tumor cells. Preventive strategies involving vaccines have been extensively employed to prevent infectious disease yet their role for cancer prevention remains limited. Conversely, interception strategies have represented important fields of investigation in BC since CVs are often more effective in very early-stage disease, such as DCIS, where tumor burden is very low and the microenvironment is more permissive [19]. In advanced disease, therapeutic vaccines face challenges from higher tumor burden and immunosuppression but can induce long-term memory against residual or recurring tumor cells [15, 16, 19].

Vaccines typically include: a. Antigen(s); b. Delivery platform; c. Adjuvant(s). BC cells express tumor-associated (TAAs) and tumor-specific antigens (TSAs), processed by antigen-presenting cells to activate CD4⁺ helper and CD8⁺ cytotoxic T cells [20]. TAAs (e.g., HER2) are overexpressed normal proteins, while TSAs/neoantigens derive from somatic mutations [21]. HER2, consistently overexpressed in a subset of BC, has been widely leveraged for CVs development due to strong immunogenicity and endogenous anti-HER2 responses [22], whereas true neoantigens are rare (< 5% of cases) [23].

Delivery platforms include cell-based formulations: whole tumor cells/lysates [24, 25], peptide/protein vaccines [26–29], viral or bacterial vectors [30], and nucleic acid–based DNA/RNA vaccines [31]. Peptide vaccines targeting HER2 have shown safety and immunogenicity, especially with Granulocyte–Macrophage Colony-Stimulating Factor (GM-CSF) [26, 29] though risks include regulatory T cell activation [32]. Vectors offer strong immuno-stimulation but raise safety concerns in immunocompromised patients [30], while gene-based vaccines are flexible and scalable but often require adjuvants and specialized delivery [31]. Dendritic cells (DCs), a type of cell based vaccine, are among the most effective, but DC-based vaccines are resource-intensive, requiring patient-specific cell collection and ex vivo manipulation [33, 34]. Dendritic cells can be considered as both a type of vaccine platform and immunologic adjuvant. In DC-based vaccines, DCs represent the vaccine vehicle itself, as they are loaded with TAA or neoantigens and administered to induce antigen-specific T cell responses. At the same time, because of their intrinsic ability to provide antigen presentation, co-stimulation, and cytokine-mediated immune activation, they also exert an adjuvant-like function. However, they should not be classified as conventional adjuvants, but rather as cellular vaccine platforms with intrinsic immunostimulatory and immunomodulatory properties.

Adjuvants are key to amplifying adaptive immune cells although novel alternative synthetic and biological ones should be developed.

To address these challenges, alternative synthetic and biologic adjuvants are being developed.

Table 1 summarizes trials of HER2-targeted CVs in BC.

**Table 1:** Current studies investigating HER2 + BC Vaccines

PREVENTION-INTERCEPTION	Trial ID	Phase	Platform	Setting	n°of pts	Intervention	Patient cohort	Target/Moiety	Outcomes	Results
	NCT02061332	I/II	Dendritic cell-based	Interception	27	HER-2 Pulsed DC	DCIS, DCIS with microinvasion, DCIS with invasive disease under 5 mm	HER 2	feasibility, safety (AEs CTCAE v3.0), efficacy (CRR) and Immunogenicity	CR: 18.5%; RD: 81.5% AEs: G1:68.5%; G2:27.8%
	NCT02636582	II	Peptide-based	Interception	13	NPS + GM-CSF vs GM-CSF	DCIS HLA-A2 +	HER 2	NPS-specific CTL response; safety (AEs CTCAE v. 4.03.); efficacy (CRR)	In NPS group: CR:11.1% RD: 88.9% AEs: G1: 93.3% G2:6.7% In GM-CSF group: G1:89.7% G2:10.3% CTLs increased in both groups
	NCT04144023	Ib	Multi-epitope peptide-based	Interception	ongoing	H2NVAC	DCIS	HER 2	Safety (AEs CTCAE version 4.0; DLT); immunogenicity; efficacy (CRR); changes in HER2 expression	NA
EARLY STAGE	Trial ID	Phase	Platform	Setting	n°of pts	Intervention	Patient cohort	Target/Moiety	Outcomes	Results
	NCT01479244	III	Peptide-based	Adjuvant	758	NPS + GM-CSF vs placebo + GM-CSF	Early node + BC with low/intermediate HER2 expression	HER2-derived peptide	DFS and OS	In NPS groups: AEs leading to discontinuation: 3.5% AEs G3: 5.9% G4:0.5% Recurrence events: 9.8% In GM-CSF: AEs leading to discontinuation:3.9% AEs G3: 5.4% G4:0.3% Recurrence events: 6.3%
	NCT01570036	IIb	Peptide-based	Adjuvant	275	NPS + GM-CSF + trastuzumab vs GM-CSF + trastuzumab	HER2-low BC	HER2-derived peptide (E75)	DFS; safety (AEs CTCAE version 4.03)	In NPS group: Local AEs: G1:97.3% G2:2.6% G3:0.2% Systemic: G1:90.9% G2:7.8% G3:1.3% In non-NPS group: Local AEs: G1:96.6% G2:3.4% G3:0% Systemic: G1:91.9% G2:6.6% G3:1.5% No significant DFS improvement overall
	NCT02297698	II	Peptide-based	Adjuvant	100	NPS + GM-CSF + trastuzumab vs GM-CSF + trastuzumab	HER2 +, high-risk, early BC	HER2-derived peptide (E75)	iDFS; DRFS; safety (AEs CTCAE version 4.03)	DFS: 84% vs 94% AEs: 92% vs 86%
	NCT00524277	II	Peptide-based	Adjuvant	180	GP2 + GM-CSF vs GM-CSF alone	Early BC with HER2 1 + to 3 +	HER2-derived peptide	DFS; safety; Immune-response	In HER2 3 + subgroup: DFS 100% vs 89.4% control at 5 years In HER2 1 +/2 + subgroup: DFS 77.1% vs 77.6% Local AEs: G1:73% vs 74% G2:25% vs 26% G3:1%vs 0% Systemic AEs: G1:68% vs 63% G2:15% vs 16% G3:1%vs 1%
	NCT00524277	II	Peptide-based	Adjuvant	298 (154 Her2pos)	AE37 + GM-CSF vs GM-CSF alone	Early BC with HER2 1 + to 3 +	HER2-derived peptide	DFS; safety; Immune-response	5-year DFS rate 80.1% vs 79.3% HER2 3 + do not benefit from AE37 vaccination Local AEs: G1:72% vs 81% G2:28% vs 19% Systemic AEs: G1:51% vs 58% G2:11% vs 12% G3:1%vs 1%
	NCT04197687	Safety run-in	Multi-epitope peptide based	Post-neoadjuvant	20	TPIV100 (or H2NVAC) + T-DM1	Early BC HER2 + residual disease post-neoadjuvant	HER2-derived peptides	Safety (DLT; AEs)	Well tolerated no DLTs
	NCT04197687	Ib/II	Multi-epitope peptide based	Post-neoadjuvant	Ongoing	TPIV100 + GM-CSF	Early BC HER2 + residual disease post-neoadjuvant	HER2-derived peptides	Safety, immunogenicity, preliminary efficacy under evaluation	NA
	NCT03384914	II	Gene-based	Post-neoadjuvant	55	WOKVAC	Early BC HER2 + residual disease post-neoadjuvant	HER2 and other oncodrivers	Immunogenicity; DFS	AEs G1-G2 9 events: 1 new primary cancer 2 local recurrence 6 metastatic disease recurrence
	NCT03384914	II	Dendritic cell-based	Post-neoadjuvant	55	DC1	Early BC HER2 + residual disease post-neoadjuvant	HER2-derived peptides	Immunogenicity; DFS	AEs G1-G2 No long-term efficacy data
	NCT05325632	II	Dendritic cell-based	Neo-adjuvant	ongoing	DC1 + Paclitaxel, Trastuzumab + Pertuzumab	Stage I-III HER2 +	HER2	Radiologic tumor response rate; Immunogenicity; RFS	NA
	NCT05232916	III	Peptide-based	Post-neoadjuvant and post-adjuvant	ongoing	GLSI-100 (GP2 + GM-CSF) vs placebo	Early BC HER2 + residual disease or high-risk pCR at surgery and who have completed both neoadjuvant and postoperative adjuvant trastuzumab based treatment	HER2-derived peptide (GP2)	IBCFS; IDFS; DDFS; OS; Quality of life (QLQ-C30 and FACT-GP5); Immune Response	80% reduction in MBC recurrence rate at 5 years GLSI-100 increase immune response both in HLA-A*02 treated and placebo arms and non-HLA-A*02 open-label arm
ADVANCED/METASTATIC	Trial ID	Phase	Platform	Setting	n°of pts	Intervention	Patient cohort	Target/Moiety	Outcomes	Results
	NCT00140738	I/II	Peptide-based	Metastatic	40	dHER2 + adjuvant AS15	mBC HER 2 +	HER 2	Safety (AEs CTCAE version 3.0); efficacy (CRR)	AEs: G1-G2; CBR (all): 30% CBR (I-line):35% CBR (II-line):26% mTTP (I-line):2.8 mo mTTP (II-line):3.4 mo
	NCT00436254	I	Gene-based	Locally advanced/Metastatic	66	pNGVL3-hICD + sargramostin (arm A:10 µg; arm B:100 µg; arm C:500 µg)	stage III-IV BC HER2 +	HER 2	Safety (AEs CTCAE v 3.0); Immune response	AEs: G1: 98% G2:44% Dose-dependent T cell responses
	NCT03632941	II	Viral-based	Metastatic	8	VRP-HER2 + pembrolizumab	mBC ER-/PR-/HER2 +	HER 2	immune-response (TILs and anti-HER2 Abs); AEs	NA
	NCT00194714	I/II	Peptide-based	Metastatic	26	HER2 peptide vaccine + trastuzumab	mBC HLA-A2 +/HER2 +	HER 2	Immune-response; safety (AEs CTCAE, Version 3.0); efficacy	AEs:G1–2: 99% Inverse correlation between vaccine-induced T cell response and serum TGF-β mPFS: 17.7mo mOS not reached
	NCT00317603	I	Cell-based	Metastatic	15	Irradiated, autologous BC cells engineered by adenoviral mediated gene transfer to secrete GM-CSF	mBC (any subtype) in II line	GM-CSF release	Safety (AEs)	AEs G1- 2; PD: 67% SD:25% NED: 8.3%
	NCT00952692	I/II	Peptide-based	Metastatic	12	dHER2 + adjuvant AS15 + Lapatinib	mBC HER 2 +	HER2	Safety (AEs CTCAE, Version 3.0); N. pts with T-cell Response; Efficacy (ORR; TTP)	Well tolerated No ORR mTTP 55 days OS at 300 days: 92%
	NCT00399529	II	Cell-based	Metastatic	22	Allogeneic GM-CSF-secreting Breast Tumor Vaccine + trastuzumab + cyclophosphamide	mBC HER 2 +	HER2	Safety; efficacy (CBR; PFS; OS); immunogenicity	Well tolerated CBR at 6 mo:55% CBR at 12 mo:40% mPFS:7 mo; mOS: 42 mo
	NCT00393783	I	Gene-based (xenogenic)	Locally advanced/Metastatic	ongoing	HER2 ECD DNA vaccine injections ± ET ± trastuzumab	Stage III-IV BC HER2 +	HER2 (ECD)	Safety (DLT); anti-tumor effect	NA
	NCT04348747	II	Cell-based	Metastatic	ongoing	anti-HER2/HER3 DCs vaccine injections + celecoxib, recombinant INFalfa-2b, rintatolimod followed by pembrolizumab	mBC TNBC or HER2 + with BMs	HER2/HER3	Efficacy (CNS ORR, CNS PFS, overall PFS, mOS); safety (AEs); immunogenicity	NA
	NCT00266110	II	Multiepitope autologous dendritic cell-based	Metastatic	17	Sargramostim + therapeutic autologous DC + trastuzumab + vinorelbine	Locally recurrent or mBC HER2 +	E75-E90	Efficacy (ORR); immunogenicity	AEs Any G: 70.6% AEs G4:11.76% ORR = 0%
	NCT04521764	I	Viral-based	Metastatic	ongoing	MV-s-NAP administered intratumorally	mBC (any subtype)	Pylori neutrophil activating protein (NAP)	Safety (DLT; AEs); efficacy (Best tumor response); immune-response	NA
	NCT03328026	II	Cell-based + TME modulator	Metastatic	ongoing	SV-BR-1-GM + INCMGA00012 vaccine injections epacadostat	mBC (any subtype)	GM-CSF-release; PD-L1; IDO	Safety (AEs); efficacy (ORR; durability of response)	NA
	NCT05378464	I	Dendritic cell-based	Metastatic	ongoing	DC1 + pepinemab + trastuzumab	mBC HER 2 +	HER2	Safety (MTD); ORR; CBR; PFS	NA

In the Table we have reported available trials, divided by settings, investigating HER2 + BC vaccines with, where available the main efficacy and safety results

*Abbreviations: ID* identifier, *n°* number, *pts* patients, *BC* breast cancer, *mBC* metastatic breast cancer, *DCIS* Ductal Carcinoma in Situ, *TNBC* triple-negative breast cancer, *HER2* human epidermal growth factor receptor 2, *DCs* dendritic cells, *NPS* Nelipepimut-S, *GM-CSF* granulocyte–macrophage colony-stimulating factor, *HLA* Human Leukocyte Antigen, *CTL* Cytotoxic T-lymphocyte, *BMs* brain metastases, *PD-L1* Programmed Death-Ligand, *IDO* indoleamine-pyrrole 2,3-dioxygenase, *VRP* alphavirus-derived replicon particles, *ER* Estrogen Receptor, *PR* Progesterone Receptor,—negative, + positive, *ECD* extracellular domain, *ET* endocrine therapy, *INFalfa* interferon alpha, *TILs* tumour-infiltrating lymphocytes, *TGF-β* Transforming Growth Factor-beta, *DLT* dose-limiting toxicity, *AEs* adverse events, *CTCAE* Common Terminology Criteria for Adverse Events, *MTD* Maximum Tolerated Dose, *CR* complete radiological response, *CR* complete response, *RD* residual disease, *CBR* clinical benefit rate, *mTTP* median time to progression, *PFS* progression-free survival, *OS* overall survival, *DFS* disease-free survival, *ORR* objective response rate, *IDFS* invasive Disease-Free Survival, *DDFS* Distant Disease-Free Survival, *RFS* Relapse-Free Survival, *CNS* Central Nervous System

**Fig. 1:**
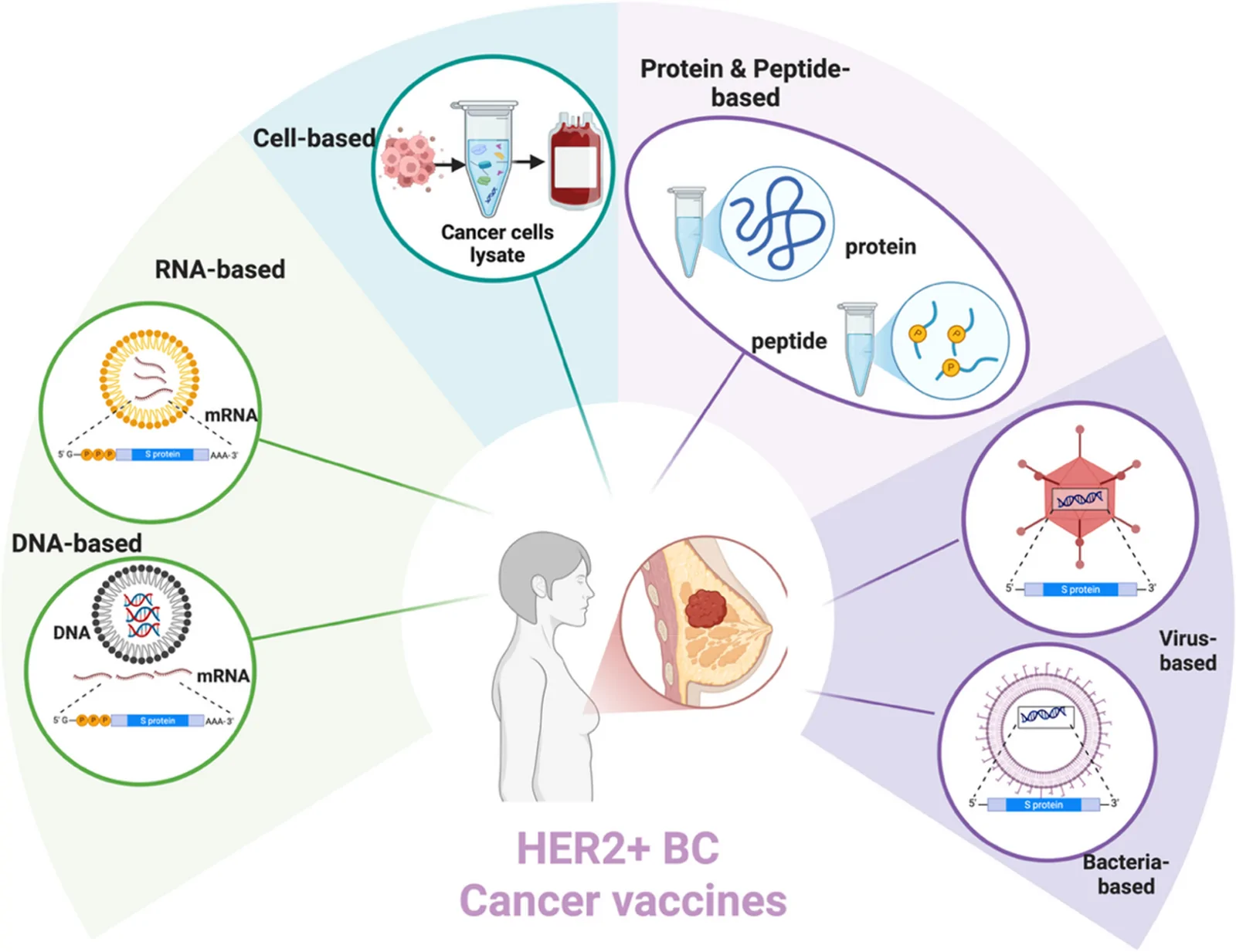
Different platforms and technologies used for cancer vaccines development. Figure 1 shows the different platforms and technologies used for cancer vaccine development: DNA-based, RNA-based, cell-based, protein/peptide-based, virus/bacteria-based

Figure 1 illustrates vaccine platforms.

Table 2 shows an overall summary of vaccine advantages, limitations and safety profile.

**Table 2:** Summary of vaccine advantages, limitations and safety profile

Vaccine type	Advantages	Limitations	Selected Clinical Trials	Safety profile
Peptide/protein-based	High antigen specificity; ease of manufacturing; reproducible immunogenicity	HLA restriction; limited efficacy as monotherapy	NPS/E75: NCT02636582, NCT01479244, NCT01570036, NCT02297698. GP2/GLSI-100: NCT00524277, NCT05232916. AE37: NCT00524277. HER2 peptide + trastuzumab: NCT00194714. dHER2 +. Adjuvant AS15 NCT00140738	Predominantly grade 1–2 AEs; injection-site reactions; flu-like symptoms
Multi-epitope peptide	Broader epitope coverage; CD4⁺/CD8⁺ activation; reduced HLA restriction	Limited phase III evidence; need for combination strategies	H2NVAC/TPIV100: NCT04144023, E75-E90 NCT04197687	Mostly grade 1–2 AEs
DNA/RNA-based	Scalable platform; flexible design; induction of humoral and cellular immunity	Low intrinsic immunogenicity; delivery limitations	WOKVAC: NCT03384914. pNGVL3-hICD: NCT00436254. HER2 ECD DNA vaccine: NCT00393783	Mild AEs; no significant long-term toxicity reported
Dendritic cell-based	Efficient antigen presentation; strong T-cell priming; Th1 polarization	Complex manufacturing; high cost; limited scalability	HER2-pulsed DC/DC1: NCT02061332, NCT03384914, NCT05325632, NCT04348747, NCT00266110, NCT05378464	Grade 1–2 AEs; fever; fatigue; injection-site reactions
Autologous cell-based	Personalized antigen repertoire; durable immune responses	Time-consuming production; high cost	Autologous GM-CSF–secreting BC cells: NCT00317603	Favorable safety; minimal grade ≥ 3 AEs
Allogeneic cell-based	Off-the-shelf availability; broad antigen exposure	Heterogeneous immune response; lower specificity	Allogeneic GM-CSF–secreting breast tumor vaccine + trastuzumab/cyclophosphamide: NCT00399529. SV-BR-1-GM: NCT03328026	Mild systemic AEs; injection-site reactions
Viral/bacterial vector-based	Strong innate and adaptive immune activation; efficient antigen delivery	Reduced efficacy due to pre-existing anti-vector immunity; potential risks in immunocompromised patients	VRP-HER2 + pembrolizumab: NCT03632941. MV-s-NAP: NCT04521764. MVA-BN/FPV-brachyury combinations: NCT04296942	Mostly mild–moderate AEs
Adjuvanted protein vaccines	Combined humoral and cellular responses; enhanced immunogenicity	Variable efficacy in advanced disease; influenced by prior therapies	dHER2 + AS15: NCT00140738. dHER2 + AS15 + lapatinib: NCT00952692	Grade 1–2 AEs; rare cardiac events (e.g., LVEF decline)

In Table 2, for each vaccine types, we summarized key advantages, limitations while citing selected clinical trials where each cancer vaccine type has been employed and the severity of adverse events reported

*Abbreviations: HER2* human epidermal growth factor receptor 2, *NPS* Nelipepimut-S, *GM-CSF* granulocyte–macrophage colony-stimulating factor, *HLA* Human Leukocyte Antigen, *AEs* adverse events, *BC* breast cancer, *ECD* extracellular domain, *VRP* alphavirus-derived replicon particles, *LVEF* Left Ventricular Ejection Fraction

### “Mechanisms of immune response induced by HER2 vaccines”

Several differentiation programs shape the direction, quality, durability, and balance of the anti-tumor immune response.

- (1a-b) Vaccine administration and antigen uptake by APCs.

Following administration, if the HER2 vaccine is based on cellular lysates, peptides, or proteins (1a), it is taken up by professional antigen-presenting cells (APCs), most likely DCs, through endocytosis or phagocytosis. In this phase, the vaccine antigen enters the endosomal compartment, where exogenous HER2-derived material is internalized for processing. If the vaccine is RNA- or DNA-based and/or delivered through viruses or bacteria (1b), this phase differs somewhat. In the case of RNA- or DNA-based vaccines, lipid nanoparticles (LNPs) protect the nucleic acid cargo and facilitate its entry into host cells, particularly DCs. Once in the cytoplasm, mRNA is translated by host-cell ribosomes into TAAs, whereas plasmid DNA must first reach the nucleus, where it is transcribed into mRNA and subsequently translated into tumor antigens. These newly synthesized antigenic proteins are then processed and presented on the cell surface through both Major Histocompatibility Complex (MHC) class I and, in some cases, MHC class II molecules. Virus and bacteria-based vaccines act as vehicles to deliver specific genetic material or proteins. Once administered these are engulfed by dendritic cells, processed, and presented on MHC molecules to T cells.

This initial step is essential because it determines how efficiently HER2 epitopes will be generated and displayed to naïve CD4 + T cells. The nature of the vaccine platform and the presence of adjuvants strongly influence APC maturation and the inflammatory milieu that will later guide T cell polarization.

- (2) Endosomal/lysosomal processing and loading onto MHC class II

The HER2 antigen traffics from early endosomes into progressively acidified endosomal/lysosomal compartments, where proteolytic enzymes degrade the antigen into immunogenic peptide fragments. These processed peptides are then loaded onto MHC class II molecules within specialized vesicular compartments. The peptide–MHC II complexes are subsequently transported to the APC surface. This step is central for activating CD4 + T helper cells, because MHC II-restricted presentation is the canonical route by which exogenous vaccine antigens are recognized. Efficient peptide generation and stable peptide–MHC II complex formation are decisive for the magnitude of helper T cell priming.

- (3) Priming of naïve CD4 + T cells: antigen recognition and co-stimulation

At the APC surface, the HER2 peptide–MHC II complex is scanned by the T cell receptor (TCR) on naïve CD4 + T cells. Productive priming requires at least two coordinated signals.

The first is antigen-specific recognition, delivered by TCR engagement of the peptide–MHC II complex whereas the second is co-stimulation, represented in the figure by the interaction between B7 molecules on the APC and CD28 on the T cell. This co-stimulatory signal promotes T cell survival, metabolic activation, IL-2 production, and clonal expansion. In the absence of adequate co-stimulation, T cells may become anergic or functionally impaired, which is particularly relevant in cancer, where tolerance and immune suppression are common barriers. Together, TCR signaling plus CD28-mediated co-stimulation initiate activation of the naïve CD4 + T cell and prepare it to respond to the surrounding cytokine environment.

- (4) Cytokine-driven polarization of activated CD4 + T cells

Once primed, the activated CD4 + T cell integrates antigenic and co-stimulatory inputs with the local cytokine milieu produced by APCs and other immune cells. These cytokines instruct lineage-specific transcriptional programs and direct differentiation into distinct CD4 + T cell subsets. Thus, the vaccine does not simply activate helper T cells; it shapes their functional identity [35, 36].

This is a critical determinant of whether the resulting anti-HER2 response will favor immune regulation, durable memory, antibody production, or cell-mediated anti-tumor immunity.

- (5) Differentiation into specialized CD4 + T-cell subsets and functional outcomes

**Fig. 2:**
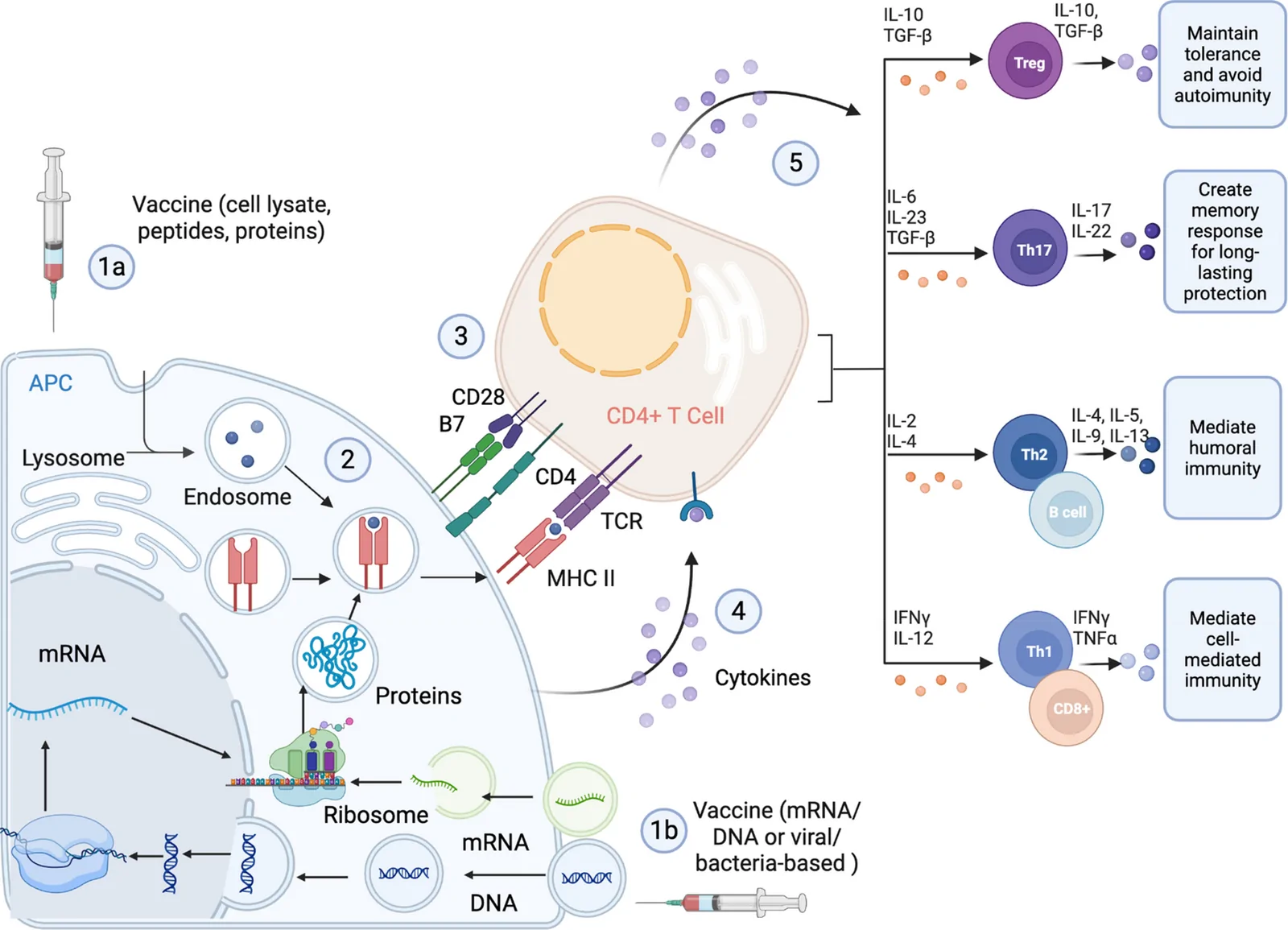
The molecular mechanisms underlying HER2 vaccine-induced immune activation. Figure 2 shows the mechanisms that orchestrate HER2 vaccine-induced immune activation: after vaccination (1), the antigen is taken up by an antigen-presenting cell (APC) and trafficked into endosomal/lysosomal compartments for processing (2). Antigenic peptides are then loaded onto MHC II and displayed on the APC surface, where a CD4⁺ T cell recognizes them through its TCR and receives co-stimulatory signaling via CD28–B7 (3). Cytokines released by the APC and surrounding environment further shape the activation and fate of the CD4⁺ T cell (4). Depending on these cytokine cues, the activated CD4⁺ T cell differentiates into distinct subsets—Treg, Th17, Th2, or Th1—that drive tolerance, long-lasting memory/protection, humoral immunity, or cell-mediated immunity, respectively (5)

Figure 2 shows HER2 + CVs immune activation and the four major polarization pathways.

#### Treg differentiation

In the presence of IL-10 and TGF-β, activated CD4 + T cells differentiate into regulatory T cells (Tregs) [37–39]. These cells are essential for preserving peripheral tolerance and limiting excessive immune activation. Their dominant function is to restrain inflammatory responses and reduce the risk of autoimmunity. In the context of HER2 vaccination, this pathway is mechanistically important because HER2 is a self-associated tumor antigen. A certain degree of regulation may help prevent off-target tissue damage; however, excessive Treg induction may also dampen vaccine efficacy by suppressing anti-tumor effector responses. Thus, the Treg axis reflects the balance between safety and immunogenicity.

#### Th17 differentiation

Exposure to IL-6, IL-23, and TGF-β promotes differentiation toward the Th17 lineage [40–42]. Th17 cells characteristically produce IL-17 and IL-22, cytokines involved in tissue inflammation, recruitment of additional immune cells, and maintenance of barrier-associated immunity. In vaccination, Th17 responses may contribute to inflammatory amplification and, in some settings, to the establishment of long-term immune memory. In the anti-tumor setting, Th17 cells can be functionally complex: they may support immune activation and foster durable responses, but under some conditions they may also contribute to chronic inflammation or functional plasticity which is linked to the concept of a memory-supportive response for long-lasting protection, highlighting the idea that inflammatory helper programs can reinforce persistence of vaccine-induced immunity.

#### Th2 differentiation

In a milieu dominated by IL-2 and IL-4, activated CD4 + T cells polarize into Th2 cells [43–45]. These cells produce cytokines such as IL-4, IL-5, IL-9, and IL-13, which are central to humoral immunity. Mechanistically, Th2 cells provide help to B cells, promoting antibody class switching, affinity maturation, and plasma cell differentiation. In the setting of a HER2 vaccine, this pathway supports the generation of anti-HER2 antibodies capable of binding the receptor on tumor cells. Such antibodies may contribute to tumor control by blocking HER2 signaling, promoting opsonization, and potentially enhancing Fc-dependent immune mechanisms. Therefore, the Th2 branch in the figure represents the helper axis most directly associated with vaccine-induced antibody responses.

#### Th1 differentiation

When the cytokine environment contains IL-12 and IFN-γ, activated CD4 + T cells differentiate into Th1 cells [36, 46]. Th1 cells produce IFN-γ and TNF-α, which are key mediators of cell-mediated immunity. This is generally the helper program most strongly associated with effective anti-tumor immune responses. Th1 cells enhance macrophage activation, support antigen presentation, promote inflammatory chemokine production, and, importantly, provide help for the generation and maintenance of CD8 + cytotoxic T cell responses. In the context of HER2-positive breast cancer vaccination, Th1 polarization is particularly desirable because it helps establish a tumor-directed cellular immune response capable of recognizing and eliminating malignant cells. The Th1 branch shown in the figure therefore represents the effector arm most closely linked to direct anti-tumor immunity.

## Trials in BC

### Prevention and interception

CVs may help prevent progression to invasive BC (IBC) by targeting pre-invasive lesions such as Ductal carcinoma in situ (DCIS) and lobular carcinoma in situ (LCIS). HER2, expressed in > 50% of DCIS but only in 20–30% [47] of invasive tumors, is an attractive early target, as immune elimination of HER2 + cells could promote tumor control. HER2 is more frequent in high-grade DCIS, linked to recurrence risk [48], and implicated in cancer pathogenesis [49, 50]. HER2 amplification often co-occurs with cytokine gene clusters inversely correlated with CD8⁺ granzyme B + T cells, key effectors of tumor clearance [51]. In HER2 + DCIS, Th1 immunity declines progressively, with near-complete loss in HER2 + IBC and advanced disease [52]. Restoring anti-HER2 Th1 responses improves survival, highlighting the value of immune targeting in early disease. Thus, HER2-directed vaccination represents a promising strategy to modify DCIS natural history and reduce recurrence.

#### Autologous cell-based vaccines

##### HER2 dendritic cell vaccine for DCIS

Brian J. Czerniecki and colleagues evaluated a DC vaccine pulsed with HLA class I/II HER2 peptides in HER2 + DCIS [52]. In a pilot study, patients received ex vivo–activated DC1s prior to surgery, leading to robust HER2-specific CD4⁺/CD8⁺ T-cell responses, tumor-lytic antibodies, and increased lymphocytic infiltration. Two of 11 patients had no residual disease, while 50% of those with residual tumors lost HER2 expression, consistent with vaccine-induced immunoediting [52]. Toxicities were limited to grade 1–2 [52].

The clinical evidence of immune activation and tumor reduction in these trials is supported by mechanistic studies in HER2/neu transgenic mouse models (e.g., BALB-neuT). Preclinical data have demonstrated that the prevention and interception of early mammary carcinogenesis are heavily driven by the deployment of highly polarized type-1 dendritic cells (DC1). When candidate DCs are activated ex vivo via conditioning with interferon-gamma and lipopolysaccharide (LPS) (a TLR4 agonist), they undergo critical maturation. This molecular licensing induces a high surface density of costimulatory molecules and maximizes the sustained secretion of Interleukin-12p70 (IL-12p70), a pivotal cytokine required to prime high-avidity, antigen-specific T cells while effectively preventing regulatory T-cell (Treg) mediated anergy [33, 49].

Furthermore, functional murine models elucidated that the direct intranodal delivery route bypasses the biological requirement for DC migration from peripheral injection sites. This targeted local–regional administration optimizes the immediate presentation of HLA class I/II HER2 peptides to naïve T cells directly within the secondary lymphoid organs. Preclinical evidence also established that vaccine-mediated protection depends crucially on the expansion of IFN-gamma-producing CD4⁺ Th1 helper cells. Rather than relying strictly on direct cytotoxic T-lymphocyte (CTL) mediated lysis, these CD4⁺ Th1 cells are indispensable for licensing and sustaining a robust humoral arm. This response is characterized by complement-fixing, tumor-lytic IgG antibodies (the murine IgG2a isotype) that work in tandem with cellular immunity to exert a rigorous selective pressure. This dual cellular and humoral attack explains the downstream clinical phenomenon of antigen-specific immunoediting and the subsequent loss of HER2 oncodriver expression observed in residual clinical lesions [49, 53].

In a subsequent phase II trial [33] (*n* = 27), weekly Immune Conditioning via Activated Innate Transfer (ICAIT) vaccination, with the same vaccine, elicited strong HER2-specific T cell responses, with 18.5% achieving pathologic complete response (pCR) [33]. Half of residual tumors lost HER2, and ER status influenced efficacy (response 90% in ER– vs 5.9% in ER +) with some tumors shifting their phenotype (e.g., ER +/HER2 + to ER +/HER2–) [33] (Table 1).

A later randomized study [54] confirmed safety across intralesional, intranodal, and combined delivery routes, with higher pCR rates in DCIS (28.6%) than IBC (8.3%) (Table 1). Notably, ER + DCIS patients receiving concurrent endocrine therapy achieved response rates comparable to ER– cases [54].

Overall, HER2-targeted DC vaccination in early-stage BC, particularly DCIS, induces durable antigen-specific immunity, lymphocyte infiltration, tumor phenotype shifts, and occasional complete responses.

#### Vaccines based on HER2-derived peptides

##### Nelipepimut-S peptide vaccine

Nelipepimut-S (NPS, also known as E75) is a nine–amino acid peptide (HER2 369–377, KIFGSLAFL) with high affinity for HLA-A2 and HLA-A3, derived from the extracellular domain of HER2 [55]. Preclinical data provide strong evidence for the immunogenicity of NPS, characterizing it as an immunodominant epitope capable of eliciting robust HER2-specific CD8⁺ CTL responses [55]. At the molecular level, as a short peptide, NPS bypasses complex intracellular processing by binding directly to MHC class I molecules on the surface of APCs [56].

Furthermore, preclinical studies have demonstrated a significant synergism between HER2-targeted peptide vaccines and trastuzumab [57]. Specifically, in vitro models show that trastuzumab facilitates the uptake of HER2 by DCs through Fc receptor-mediated endocytosis, which significantly increases the cross-presentation of HER2-derived peptides (including NPS) to CD8 + T cells [56].

This synergy suggests that trastuzumab not only blocks HER2 signaling but also acts as an immune adjuvant by enhancing the visibility of tumor cells to vaccine-induced CTLs. Based on this rationale, NeuVax, a formulation of NPS plus GM-CSF, was evaluated in HLA-A2–positive women with DCIS in a randomized trial [58] comparing the vaccine versus GM-CSF alone before surgery.

During the first month, NeuVax recipients showed a numerically greater—but not statistically significant—increase in circulating NPS-specific CTLs. Histologic analysis demonstrated changes in tumor-infiltrating lymphocytes (increased iTILs and reduced sTILs from biopsy to surgery), though differences between treatment arms were not significant [58]. Since iTILs are established predictors of pCR and favorable long-term outcomes in early HER2 + disease [59], these findings suggest vaccination may modulate the tumor microenvironment in a clinically relevant way (Table 1).

Interestingly, both arms exhibited a sustained rise in circulating NPS-specific CTLs, raising the possibility that GM-CSF alone may stimulate immunity against HER2. No clear differences were observed in residual DCIS, lymphocytic infiltration, or HER2 expression [59]. The trial was limited by small enrollment, which reduced statistical power which hindered correlative analyses.

In summary, this study provides a rationale for further testing of HER2 peptide vaccines in the (neo)adjuvant DCIS setting. The observed immune activity, coupled with challenges of accrual and trial design in prevention, underscores both the potential and the hurdles for HER2-targeted vaccination strategies.

##### H2NVAC

H2NVAC is a multi-epitope peptide vaccine comprising four immunogenic sequences derived from the HER2 TAA. This vaccine is designed to elicit a robust helper T cell–mediated immune response against HER2-overexpressing tumor cells. By engaging CD4⁺ T cells, H2NVAC aims to promote sustained immune activation and amplify anti-tumor immunity through enhanced T helper cell support.

A phase Ib clinical trial (NCT04144023 and Table 1) is currently evaluating the safety, tolerability, and optimal dosing of H2NVAC when administered preoperatively in patients with HER2-expressing DCIS. The trial seeks to determine whether H2NVAC can effectively stimulate tumor-specific T cell responses, with the ultimate goal of enhancing immune-mediated clearance of pre-invasive lesions and preventing progression to IBC.

#### Challenges and recommendations in the interception/prevention Setting:

Implementing vaccines for cancer interception and primary prevention faces substantial methodological and clinical hurdles that warrant careful consideration.

A primary obstacle lies in target and biomarker identification; the relative paucity of endogenous tumor antigens at the pre-cancer stage can severely impair effective T cell priming. This challenge is further compounded when pre-cancerous tissue is unavailable for genomic sequencing, making it exceptionally difficult to identify reliable surrogate biomarkers capable of predicting long-term clinical benefits, such as the prevention of invasive disease. Moreover, in healthy cohorts lacking established genetic susceptibility factors, current oncology clinics possess no predictive tools to anticipate either an individual’s specific oncogenic trajectory or potential future TSAs.

Beyond antigen selection, vaccine platforms themselves present significant translational barriers.

While highly immunogenic platforms like viral, bacterial, or dendritic cell-based vectors have achieved success in advanced therapeutic regimens, their deployment in an interception framework is heavily restricted.

Targeting otherwise healthy populations demands stricter safety profiles and an exceptionally high risk–benefit threshold, which limits the use of these more aggressive platforms. Finally, mitigating these challenges requires a concerted effort to enhance patient and provider education regarding the safety profiles of preventive cancer vaccines. 

### Early-stage BC

In contrast with advanced stage [2], early BC presents a more favorable immunologic milieu for vaccination. Multiple clinical studies have shown that immunotherapy, such as immune checkpoint inhibitors (ICIs), especially in triple-negative BC (TNBC), can synergize with neoadjuvant or adjuvant chemotherapy in this context [60, 61], likely due to preserved immune competence, limited tumor burden, and reduced cumulative toxicity from prior treatments. These conditions not only help maintain host immunocompetence but also mitigate mechanisms of immune escape and acquired resistance [62]. Consequently, although ICIs has not replicated in HER2 + disease [11] the same benefit of TNBC, early-stage disease is increasingly recognized as the optimal setting for a novel class of immunotherapy, such as CVs, development, where intact immune function, lower clonal heterogeneity, and a less immunosuppressive TME collectively enhance the likelihood of a durable therapeutic response.

Furthermore, it has been proven that also in early stages the TME might favor tumour progression through the dissemination of cancerous cells which are known as metastasis-initiating cancer cells (MICs). Since MICs are intrinsically resistant to the available therapies and are the source of tumour metastases, several efforts have been made to try to inhibit MICs growth by acting on TME with the help of CVs. Indeed, intratumoral delivery of type I polarized DC1 limited the MIC dissemination mechanism in HER2 + BC through the secretion of INF-γ. Initial reports suggested a reduced burden of MICs in the bone marrow of patients with stage I–III HER2 + BC treated with intratumoral DC1, compared with patients treated with standard neoadjuvant HER2 regimens [63].

These findings indicate an intriguing strategy to inhibit the dissemination of MICs and to prevent the genesis of metastatic disease in patients with early BC.

#### Vaccines based on HER2-derived peptides

##### Nelipepimut-S peptide vaccine

E75 (KIFGSLAFL) has also been tested in early-stage BC [54]. Although early-phase studies suggested promising immunologic activity, these results were not confirmed in a pivotal phase III trial [55]. This study enrolled node-positive, early-stage patients with low to intermediate HER2 expression, randomized to adjuvant NPS + GM-CSF versus placebo + GM-CSF. The trial was stopped early for futility after showing no improvement in disease-free survival (DFS) [55]. Preclinical evidence of synergy between NPS, GM-CSF, and trastuzumab provided the rationale for a subsequent phase IIb study [64]. Patients received one year of trastuzumab, then they were randomized to NPS + GM-CSF or GM-CSF alone. While no overall DFS benefit was observed in HER2-low disease, subgroup analyses suggested benefit among HLA-A24 carriers and patients with triple-negative BC [64]. Further evaluation came from a randomized phase II trial (NCT02297698) in high-risk, HER2-overexpressing (IHC 3 +), non-metastatic patients [65]. Participants received trastuzumab with or without NPS. The combination was safe but failed to improve efficacy endpoints: iDFS was 79% in the NPS arm versus 92% with placebo, and DRFS was 90% versus 95%, respectively [65] (Table 1).

##### GP2

GP2, derived from the transmembrane domain of HER2, is an HLA class I–restricted peptide designed to elicit CD8⁺ cytotoxic T cell responses. In a phase IIb trial, a GP2 + GM-CSF vaccine showed favorable outcomes versus GM-CSF alone in women with operable BC across all HER2 expression level [66]. In the subgroup with HER2-overexpressing tumors (IHC 3 +), no recurrences occurred in the vaccine arm after a median of five years, translating into a 100% DFS compared with 89.4% in controls [66] (Table 1). This corresponds to an estimated ≥ 80% reduction in recurrence risk- substantially greater than the 20–50% reductions typically achieved by approved agents in this setting.

In preclinical models HER2 protein sequence was screened for peptides predicted to bind HLA-A2 based on anchor residues, leading to the identification of GP2, a peptide from the transmembrane domain of HER2 expressed in breast cancer. In vitro preclinical studies: HLA-A2-positive breast cancer TIL lines recognized GP2 in cytotoxicity assays GP2-sensitised CTLs lysed HLA-A2 +/HER2 + breast tumour cells. These studies confirmed HER2 as a TAA and GP2 as an immunogenic peptide recognized by the endogenous immune system in breast cancer. Following GP2 identification in breast cancer, other groups demonstrated GP2 epitope presence in additional epithelial tumors [67].

On this basis, GP2 + GM-CSF (GLSI-100) is under evaluation in the Flamingo-01 trial [68], a prospective, randomized, double‑blind multicenter phase III trial (NCT05232916). The trial enrolling patients with HER2 3 + BC and either residual disease or high-risk pCR after neoadjuvant and adjuvant anti-HER2 therapy. Participants receive six intradermal doses over six months and five boosters over 2.5 years (total treatment 3 years plus 1 year follow‑up). Preliminary data indicate that GLSI-100 induces progressively stronger immune responses with repeated vaccination in both HLA-A02–positive (vaccine vs. placebo) and HLA-A02–negative (open-label) cohorts [60]. Interestingly, baseline GP2-specific immune responses were also detected in both arms, suggesting that GP2 may act as a natural antigen, potentially primed during prior trastuzumab or other HER2-directed therapies [69]. Recent preliminary immune-response data was presented: in this analysis of 247 vaccinated non‑HLA‑A*02 patients, injection‑site reactions (ISRs) — used as an in vivo marker of immune activation and assessed 48–72 h post‑injection — increased significantly from 20.2% after the first dose to 55.3% after the fourth to sixth doses (McNemar p < 0.001). Further analyses will explore correlations between delayed-type hypersensitivity test (DTH) and ISRs, HLA‑specific immunogenicity, booster timing, site performance and management of non‑responders [70].

##### AE37

AE37 is designed to stimulate CD4⁺ helper T cell responses by targeting the intracellular domain (ICD) of HER2. Unlike class I–restricted vaccines, AE37 incorporates a MHC class II epitope linked to the Ii-Key peptide (LRMK), which enhances antigen presentation and promotes robust helper T cell–mediated immunity. AE36 is a HER2‑derived MHC class II peptide (residues 776–790; Ac‑GVGSPYVSRLLGICL‑NH2) that elicits peptide‑specific CD4 + T‑cell responses in breast and ovarian cancer patients. AE37 comprises the AE36 sequence covalently linked to the four‑amino‑acid Ii‑Key motif (LRMK) from the invariant chain. The Ii‑Key motif promotes extracellular, allosteric loading of MHC class II molecules—bypassing conventional intracellular processing—and can increase epitope binding potency by up to ~ 250‑fold, favoring a more receptive conformation of the binding groove.

In preclinical models, immunization with the Ii‑Key–AE36 hybrid markedly enhanced CD4 + T‑cell proliferation to the native peptide, increased IFN‑γ secretion and augmented CTL antitumor activity. Human CD4 + T cells primed with Ii‑Key–AE36 provided substantially stronger helper support to autologous HER2‑specific CD8 + T cells, as shown by IFN‑γ ELISPOT and tumour‑cell lysis assays. These findings supported clinical development of the AE37 vaccine [71, 72].

In a phase II trial, patients with node-positive or high-risk node-negative BC, regardless of HER2 expression level, were randomized to AE37 + GM-CSF or GM-CSF alone [73]. No significant difference in DFS was observed between study arms (Table 1). However, exploratory subgroup analyses suggested possible benefit in patients with HER2 under-expression (IHC 1–2 +), advanced-stage disease, or TNBC. These signals raise the prospect that AE37 may hold value where standard HER2-targeted therapies are less effective or in combination strategies with other immunomodulators.

##### TPIV100

TPIV100 is a multi-epitope peptide vaccine composed of four HER2/neu-derived peptides targeting distinct immunogenic regions of HER2. Immunogenicity is measured using IFN-γ ELISpot analysis, and correlative studies include T cell response, HER2-specific antibody responses, and HLA genotype.

By broadening epitope coverage across diverse HLA haplotypes, it aims to enhance both immunomodulatory and antitumor activity. To boost immunogenicity, the vaccine is co-administered with sargramostim (GM-CSF), which is also a dendritic cell activator that facilitates antigen presentation and amplifies T cell responses [68].

In a recent safety run-in trial [74], patients received six cycles of TPIV100 (also called H2NVAC) with T-DM1, followed by booster doses at 3 and 12 months (Table 1). The regimen was generally well tolerated: most treatment-related adverse events (TRAEs) during the DLT period were grade 1–2, including fatigue, peripheral neuropathy, and injection-site reactions. After the DLT window, four patients experienced grade 3 AEs possibly related to the vaccine, but no new safety concerns emerged.

Based on these results, a multicenter, randomized, placebo-controlled phase II trial (NCT04197687) is now ongoing to assess the efficacy and immunogenicity of H2NVAC versus placebo in combination with T-DM1 for patients with residual disease following neoadjuvant trastuzumab and pertuzumab. This population is at particularly high risk: residual disease after neoadjuvant therapy is a well-established marker of poor prognosis in HER2 + BC [75]. While adjuvant T-DM1 improves outcomes [76], ~ 12% of patients still relapse, highlighting the need for additional strategies.

#### DNA-based and dendridic cell-based vaccines

##### WOKVAC and DC1

Two additional HER2-directed vaccine strategies have been tested in the post-neoadjuvant setting for patients with residual HER2 + BC: the multivalent DNA vaccine WOKVAC and the DC–based vaccine DC1 [77]. Eligible patients were stratified by Residual Cancer Burden (RCB 1–2 vs. RCB 3) and randomized to receive either vaccine in the adjuvant setting.

- WOKVAC is a DNA vaccine encoding multiple oncodriver antigens, including HER2, designed to elicit both CD4⁺ and CD8⁺ T cell responses through in vivo epitope expression.
- DC1 is an autologous DC vaccine, pulsed ex vivo with HER2 peptides and polarized toward a Th1 phenotype using IFN-γ and LPS, to enhance antigen presentation and helper T cell–mediated immunity.

Both platforms demonstrated favorable safety. TRAEs were limited to grade 1–2 events: in the DC1 arm, the most common were injection-site reactions, chills, fever, and fatigue; in the WOKVAC arm, injection-site reactions and fatigue predominated [77]. Preliminary efficacy signals included one case of a new primary cancer, two local recurrences, and six metastatic recurrences (Table 1) [77].

Although not powered to compare survival outcomes, this study showed that both WOKVAC and DC1 are safe, feasible, and well tolerated in the adjuvant setting after neoadjuvant HER2-directed therapy, supporting further development of vaccine-based approaches in high-risk HER2 + disease.

#### Challenges and recommendations in early stage

Despite an overall favorable safety profile, these trials reveal several methodological limitations and recurrent challenges in the clinical translation of CVs. Most studies are confined to phase I or II, with small, heterogeneous cohorts and variable efficacy end‑points, reflecting persistent obstacles in vaccine development. Although early‑phase trials of peptide vaccines such as E75 have demonstrated immune activation and reduced recurrence in phase II, no breast‑cancer vaccine has met primary survival endpoints in phase III, and results have generally shown minimal benefit beyond standard therapies. Moreover, the typically small sample sizes of phase I studies (often < 50 participants) limit statistical power and the generalizability of findings.

### Advanced/metastatic BC

The advent of HER2-targeted therapies has substantially improved outcomes in HER2 + metastatic BC (MBC), yet disease progression remains inevitable. Clinical trials of HER2-directed vaccines in this setting have so far shown limited benefit, likely reflecting the immunologically unfavorable milieu of advanced disease. Patients with MBC are often heavily pretreated, harbor high tumor burden, and experience systemic immunosuppression, all of which constrain the ability to mount effective vaccine-induced responses [78].

Beyond host factors, the metastatic TME itself poses formidable barriers. Immune escape mechanisms such as HLA class I downregulation, defects in antigen processing machinery, and reduced HER2 expression diminish tumor immunogenicity. In parallel, metastatic sites often evolve into “cold” TMEs enriched with suppressive cytokines, myeloid-derived suppressor cells (MDSCs), and regulatory T cells, further attenuating anti-tumor immunity [16, 79].

#### Protein-based vaccines

##### dHER2 with the immunostimulant AS15

The dHER2 vaccine is a recombinant fusion protein comprising the HER2 ECD and a truncated ICD, specifically excluding the kinase domain to prevent cross-reactivity. Preclinically, this construct elicited robust CD4 +/CD8 + T cell and polyclonal antibody responses that suppressed HER2 + tumor growth. Mechanistically, the ECD aims to mimic trastuzumab-mediated receptor degradation, while the ICD promotes cellular immunity through epitope spreading [80].

A phase I/II study (NCT00140738) dHER2 with the immunostimulant AS15 in patients with HER2-overexpressing MBC as first- or second-line therapy after response to trastuzumab [78]. This strategy aimed to induce both T cell–mediated and polyclonal antibody responses, potentially offering broader and more durable anti-tumor effects than monoclonal antibodies alone.

The vaccine was generally well tolerated: most adverse events were grade 1–2 (muscle pain, diarrhea, arthralgia), with occasional grade 3 events. Five patients developed asymptomatic declines in LVEF [81]. Clinically, one first-line patient achieved a complete response, one second-line patient a partial response, and 12 had stable disease, yielding a measurable clinical benefit rate and median time to progression (Table 1).

Immunologically, robust humoral responses were observed, particularly in trastuzumab-naïve patients. However, contrary to preclinical, anti-HER2 ECD antibody responses were modest and short-lived, strong and sustained responses targeted the HER2 ICD. This unexpected immunodominance of the ICD was most evident in trastuzumab-naïve patients, whereas many pretreated patients already had baseline anti-HER2-ECD antibodies likely induced by prior trastuzumab exposure, complicating interpretation of vaccine-induced responses.

In summary, the dHER2/AS15 vaccine was safe, immunogenic, and associated with clinical benefit, particularly in trastuzumab-naïve patients. These results support continued evaluation of protein-based HER2 vaccines, especially in earlier-stage disease or less heavily pretreated populations.

##### HER2/neu-specific vaccination + trastuzumab

Since the TME can suppress CD4 + and CD8 + T cell anti-tumor activity, allowing HER2 + BC cells to evade immune surveillance recent immunotherapeutic strategies have also focused on improving HER2-targeted treatments by coupling them with antibodies to enhance clinical response [82].

Indeed, preclinical studies conducted in mice have investigated the possibility of delivering intratumoral multiepitope MHC-II HER2 peptides-pulsed type I polarized dendritic cells (HER2-DC1) in combination with anti-HER2 antibodies. The combination resulted in high complete tumour regression (75–80% of treated mice) and in an increase in tumor infiltrating lymphocytes (CD4 + and CD8 +) and strong anti-HER2 responses [83].

These findings provide preliminary evidence that HER2 + CVs in combination with anti-HER2 antibodies can be tested as an effective alternative therapeutic strategy and prompt studies in humans. A phase I/II study (NCT00194714) investigated the safety and immunogenicity of combining trastuzumab with a HER2/neu-specific T-helper peptide vaccine in patients with HER2-overexpressing MBC [84]. Eligible patients had stable disease or complete remission on trastuzumab at enrollment.

The regimen was generally well tolerated: most adverse events were grade 1–2, including mild injection-site reactions and flu-like symptoms. Four grade 3 events occurred (injection-site ulceration, fainting, and pronounced local reactions). Cardiac toxicity was limited, with three cases of asymptomatic LVEF decline [84].

Immunologically, the combination induced strong and sustained HER2-specific T cell responses, with evidence of epitope spreading to other HER2 regions and unrelated tumor-associated antigens [84]. Vaccine-primed T cells secreted IFN-γ and recognized multiple tumor epitopes, indicating functional relevance within the TME. Notably, the magnitude of vaccine-induced T cell responses was inversely correlated with serum TGF-β levels, suggesting that vaccination may help counteract host immunosuppressive signaling.

Although follow-up was still ongoing, median overall survival had not yet been reached, raising the possibility of durable clinical benefit (Table 1).

##### dHER2 vaccine + lapatinib

Preclinical and clinical evidence [85] demonstrates that dual HER2 inhibition—combining the monoclonal antibody trastuzumab with the tyrosine kinase inhibitor lapatinib—synergistically enhances apoptosis and blocks tumor growth in vivo by more effectively neutralizing downstream signaling. Mechanistically, murine models indicate that HER2-specific polyclonal antibody responses, such as those elicited by the dHER2 vaccine, promote superior receptor internalization and degradation compared to monoclonal therapy, providing a strong rationale for combining active immunization with lapatinib to overcome resistance [85]. Building on this, a phase I trial (NCT00952692) evaluated the dHER2 (comprising both the HER2 ECD and ICD) with the adjuvant AS15 plus lapatinib in patients with trastuzumab-resistant MBC HER2-overexpressing [86].

All participants developed anti-HER2 ICD antibodies, demonstrating that immune tolerance could be overcome even in heavily pretreated patients. However, ECD-specific responses were more difficult to evaluate due to pre-existing antibodies from prior trastuzumab exposure; only three patients without recent trastuzumab treatment developed measurable vaccine-induced ECD antibodies [86].

Some patients progressed rapidly before vaccine-induced responses could fully develop. Moreover, a decline in ECD antibody titers was observed early during treatment, especially in those with recent trastuzumab exposure, likely reflecting waning drug levels [86] (Table 1). These observations suggest that continued trastuzumab coverage during vaccine priming may be necessary to maintain HER2 blockade until endogenous antibody responses mature.

In summary, while this combination induced biologic activity through ICD-specific antibody responses, clinical efficacy remained limited. The findings highlight both the challenges of vaccination in the metastatic setting and the potential value of concurrent HER2-targeted therapy during early vaccine priming.

#### DNA-based vaccines

##### Plasmid DNA encoding HER2/neu together with low doses of GM-CSF and IL-2

This strategy employs a plasmid encoding a full-length, signaling-deficient HER2 oncogene delivered with low doses of GM-CSF and IL-2 to enhance immune priming. Preclinical studies in murine models have shown that this full-length construct circumvents immune tolerance by presenting a broad array of epitopes to both MHC class I and II pathways [87]. Mechanistically, the co-administration of low-dose cytokines is designed to optimize this process: GM-CSF acts as a potent chemoattractant for dendritic cells at the injection site, while IL-2 provides essential signals for the expansion and survival of activated T cell clones.

Translating this approach, a pilot study [87] evaluated this vaccine in patients with HER2-expressing MBC receiving trastuzumab. Two of eight participants discontinued early—one due to progression and one due to erysipelas. The regimen was otherwise well tolerated. Although no immediate CD4⁺ T cell proliferation or HER2-specific cytokine release was observed, long-term follow-up revealed delayed but durable MHC class II–restricted T cell responses, and HER2-specific antibodies remained detectable for years, suggesting sustained humoral immunity [87].

A subsequent non-randomized phase I trial (NCT00436254) tested a plasmid vaccine encoding the HER2 ICD at escalating doses in patients with stage III–IV HER2 + BC [88]. The intradermal vaccine was well tolerated; most adverse events were grade 1–2, including injection-site reactions, fatigue, and flu-like symptoms. Immunogenicity analyses showed dose-dependent T cell responses, with the strongest activity in the higher-dose arms. Persistence of DNA at the injection site correlated with weaker responses, suggesting efficient local antigen clearance may be critical for optimal immunogenicity. Importantly, concurrent trastuzumab did not diminish vaccine-induced immunity.

The vaccine also promoted an expansion of central memory T cells, particularly at higher doses, and elicited robust HER2 ICD-specific T cell responses. Long-term follow-up revealed no delayed toxicities. While not powered for efficacy, patients in the highest-dose cohort demonstrated the most favorable overall and progression-free survival outcomes [88] (Table 1).

#### Autologous cell-based vaccines

##### Lapuleucel-T (APC8024) 

Lapuleucel-T is an autologous active cellular immunotherapy generated from peripheral blood mononuclear cells (PBMCs), including antigen-presenting cells (APCs), which are activated ex vivo with BA7072—a recombinant fusion protein incorporating HER2 ECD and ICD sequences linked to GM-CSF.

The fusion of HER2 domains to GM-CSF ensures targeted uptake and processing of the antigen by APCs via the GM-CSF receptor, while the inclusion of both ECD and ICD sequences provides a broad spectrum of MHC-restricted epitopes. This ex vivo process is designed to overcome immune self-tolerance to HER2. In vitro models have demonstrated that this activation leads to the efficient maturation of APCs, which then present HER2 peptides to both CD4 + and CD8 + T cells, creating a robust link between innate and adaptive immunity [89].

A phase I trial evaluated Lapuleucel-T in patients with HER2-overexpressing MBC [89]. The therapy was well tolerated, with no grade 3–4 adverse events reported (Table 1). Immunologically, it induced strong antigen-specific responses, as shown by lymphocyte proliferation and IFN-γ secretion following exposure to the immunizing antigen and HER2-derived peptides, confirming vaccine-driven T cell activation.

Clinically, one patient achieved a partial response and three experienced stable disease, suggesting potential disease-modifying activity in a subset of patients (Table 1) [89].

In summary, Lapuleucel-T demonstrated safety, immunogenicity, and the capacity to elicit durable HER2-specific T cell responses in advanced HER2⁺ BC. These findings highlight autologous cellular immunotherapy as a promising strategy that merits further investigation.

#### Allogeneic cell-based vaccines

##### Allogeneic GM-CSF-secreting breast tumor vaccine + cyclophosphamide + trastuzumab

A phase II trial (NCT00399529) evaluated a fixed-dose allogeneic GM-CSF–secreting breast tumor cell vaccine in combination with low-dose cyclophosphamide and trastuzumab in patients with HER2⁺ MBC [90]. The rationale was to enhance antigen presentation and T cell priming via allogeneic tumor antigens and GM-CSF secretion, while low-dose cyclophosphamide depleted T regs to create a more favorable immunologic milieu.

The regimen was generally well tolerated, with adverse events limited to injection-site reactions and systemic symptoms such as fatigue, urticaria, pruritus, and fever. The vaccine demonstrated immunologic activity and early signs of clinical benefit (Table 1) [90]. HER2-specific delayed-type hypersensitivity (DTH) responses occurred in 35% of patients, correlating with trends toward longer PFS and OS. Vaccine-induced CD8⁺ T cell responses increased over successive cycles, and the emergence of polyfunctional T cells was linked to more favorable clinical outcomes. Importantly, patients who experienced declines in circulating MDSCs after vaccination showed prolonged PFS and OS, suggesting that modulation of the suppressive myeloid compartment may contribute to efficacy [90].

In summary, this study showed that allogeneic tumor vaccines can be safely combined with immune modulators and targeted therapy, eliciting HER2-specific immune responses with potential clinical relevance in metastatic disease. Further trials are needed to confirm efficacy, refine combination strategies, and validate predictive immune biomarkers.

#### Challenges and recommendations in advanced/metastatic setting

The clinical translation of vaccines into the metastatic setting is hindered by significant biological and methodological barriers. A primary limitation is the high clonal heterogeneity and the risk of antigenic escape, where advanced tumors evade immune recognition by downregulating HER2 or MHC class I molecules [78, 81]. This evasion is further facilitated by a profoundly immunosuppressive tumor microenvironment, characterized by the accumulation of myeloid-derived suppressor cells and elevated levels of TGF-β, which has been shown to inversely correlate with the magnitude of vaccine-induced T-cell responses [84, 90]. Furthermore, in patients with a high tumor burden, clinical progression may occur too rapidly for the vaccine to elicit a mature and protective immune response.

To address these challenges, future strategies should prioritize vaccination in earlier lines of treatment or in minimal residual disease settings, where the immune system remains more functional and less exhausted [84, 87]. Methodological improvements should focus on synergistic combinations, such as using low-dose cyclophosphamide to deplete regulatory T cells [90] and maintaining trastuzumab coverage during the immunization phase. Finally, evaluating efficacy should move beyond traditional radiological criteria to integrate immune-related response criteria and overall survival, as these parameters better capture the delayed but durable clinical benefits characteristic of immunotherapy.

## Optimization of vaccine trial endpoints and immune monitoring

Although only one CV has reached the market to date, most vaccine candidates remain in pre‑marketing clinical development, making rigorous trial design essential. Safety monitoring must be thorough, but emphasis should shift increasingly towards clinically meaningful endpoints: prolonged survival remains the most compelling demonstration of vaccine efficacy. Early, small-scale studies reported modest signals of benefit but frequently lacked randomized, controlled designs; therefore, definitive assessment requires large phase III randomized trials using gold‑standard endpoints such as progression‑free, relapse‑free and overall survival. Immunological readouts are also critical for mechanistic insight and include ELISpot, tetramer/peptide–MHC flow cytometry, intracellular cytokine staining, bulk and single‑cell TCR sequencing, and bioinformatics approaches (for example MANAFEST) to track antigen‑specific clonal expansion. Longitudinal sampling — including circulating tumour DNA and post‑treatment tumour biopsy with assessment of residual tumour and CD3 + infiltration — and emerging spatial and single‑cell technologies will refine evaluation of epitope spreading and tumour microenvironment changes. Integrating robust clinical endpoints with comprehensive immune monitoring will accelerate regulatory approval and clarify the true therapeutic potential of cancer vaccines [91].

## Future perspectives

So far, BC vaccines have not shown consistent benefit in phase III trials. Multiple factors likely underlie this gap, including comtable 2plex manufacturing, immune tolerance, clonal resistance, and an immunosuppressive TME. A major limitation is that vaccine-induced immune responses are often transient, declining over time and failing to translate into durable survival benefit. This attenuation reflects both tolerance to TAAs [92, 93] and TME-mediated immunosuppression, the mechanisms of which remain incompletely understood [94].

To overcome current limitations future strategies are increasingly focused on five major domains:

- a) Innovative Vaccine Platform; b) New Adjuvants; c) Next-Generation Delivery Systems; d) Neoantigen Discovery and Epitope Optimization; e) New Combinations.

In Table 3 we reported current strategies to improve HER2 + BC vaccines development.

**Table 3:** Current strategies to improve HER2 + BC vaccines

Domain	Strategy	Description	Clinical Trial/Reference
Innovative Vaccine Platform	Innovative Peptide Vaccines	Tailored peptide vaccines (e.g., E75, GP2, AE37) based on HER2 expression and HLA subtype; includes chimeric B-cell peptide vaccines	E75, GP2, AE37; Chimeric B-cell vaccine [54, 58, 61, 77]
	Viral Vector-Based Vaccines	Uses MVA-BN and FPV vectors expressing HER2 antigens; induces broad immunity; being evaluated in combination with immunotherapies	NCT04296942: MVA-BN/FPV + M7824 ± T-DM1/Entinostat
	Alphavirus-Based Vaccines	VRP-HER2 vaccines utilize self-replicating RNA; enhance innate and adaptive immunity; combined with pembrolizumab in trials	NCT03632941: VRP-HER2 + pembrolizumab
New Adjuvant	Novel Molecular Adjuvants	Includes TLR agonists (e.g., MPLA), STING agonists, QS-21, and CD40 agonists to stimulate robust APC and T cell responses	MPLA (approved) [40]; STING [39], QS-21, CD40 agonists [85]
	Cytokine-Based Adjuvants	Cytokines such as IL-2, IL-12, IFNs, and GM-CSF enhance vaccine efficacy by promoting DC activation and T cell expansion	IL-2 [72, 86]], IFN [87], IL-12 [95], GM-CSF [57, 58, 72, 96]
Delivery Systems	Cell-Penetrating Peptides (CPPs)	CPPs like S4 domain enhance antigen uptake and cross-presentation, improving T cell activation and vaccine potency	S4 domain conjugates [91, 92]
Neoantigen and Epitope Optimization	HER2Δ16-Targeted Vaccines	Targeting HER2Δ16, an oncogenic isoform, enhances CD8 + T cell infiltration	HER2Δ16 vaccine + αPD-1 checkpoint blockade [97]
Combination Strategies	Vaccines + Immune Checkpoint Inhibitors	Combining vaccines with anti–PD-1 or other agents overcomes TME suppression and enhances immune durability	Preclinical and clinical combo trials [3, 80]
	HER2Δ16-Targeted Vaccines	Targeting HER2Δ16, an oncogenic isoform, enhances CD8 + T cell infiltration and synergizes with checkpoint blockade	HER2Δ16 vaccine + αPD-1 checkpoint blockade [97]
	Vaccine-Primed T Cell Expansion	Priming with HER2 vaccine plus IL-2/IL-12 supports ex vivo expansion and persistence of HER2-specific T cells	HER2 vaccine + IL-2/IL-12 for T cell expansion [98]

The Table summarizes innovative vaccine platforms, novel adjuvants, delivery systems, epitope-optimization approaches, and combination strategies designed to improve antigen presentation, strengthen HER2-specific immune responses, and overcome tumor microenvironment–mediated immune suppression

*Abbreviations: HER2* human epidermal growth factor receptor 2, *VRP* alphavirus-derived replicon particles, *RNA* RiboNucleic Acid, *TLR* Toll-Like Receptor, *STING* Stimulator of Interferon Genes, *APC* Antigen-Presenting Cell, *MPLA* Monophosphoryl Lipid A, *INF* interferon, *IL* Interleukin, *DC* dendritic cells, *GM-CSF* granulocyte–macrophage colony-stimulating factor, *CPPs* Cell-penetrating peptides, *PD-1* Programmed cell Death protein 1, *TME* Tumor Microenvironment

- Innovative Vaccine Platform

Vaccine platforms should be tailored to tumor characteristics; for instance, the E75 peptide is most effective in HER2-low tumors, particularly in TNBC disease [57, 93], while GP2 demonstrates greater efficacy in HER2-overexpressing cancers, especially when combined with trastuzumab [66, 73]. MHC class II–targeted vaccines such as AE37 may hold promise for HER2-low and TNBC disease and/or for specific HLA subtypes [73].

Emerging peptide-based vaccines continue to be evaluated. A recent phase I trial [99] investigated the safety and biological activity of two novel chimeric B cell peptide vaccines derived from trastuzumab-like (aa 597–626) and pertuzumab-like (aa 266–296) HER2 epitopes in patients with advanced solid tumors. Among 49 heavily pretreated patients, 14 achieved stable disease and two achieved a partial response. Importantly, the vaccine was well tolerated. These results support continued development in less pretreated populations.

Two notable viral vector–based vaccines strategies are currently under investigation for HER2 + BC and TNBC: a priming vaccine, a Modified Vaccinia Ankara–Bavarian Nordic (MVA-BN)-brachyury, which primes the immune system broadly against oncogenic pathways; and a booster vaccine, Fowlpox virus–brachyury (FPV-brachyury), used to sustain and amplify the T cell response [97]. After they have proven to be effective in murine models of BC [97] when combined to other agents such as T-DM1 and bispecific antibodies this strategy is under investigation in humans in a phase 1 trial (NCT04296942) both with a bifunctional antibody (M7824) targeting PD-L1 and TGF-B and T-DM1 with or without entinostat.

Beyond traditional viral and mRNA vectors, circular RNA (circRNA) is emerging as a robust platform for cancer immunotherapy due to its superior stability and resistance to exonuclease degradation. Recent research has demonstrated that circRNA can be engineered to ensure prolonged and stable expression of therapeutic payloads; for instance, a circRNA platform encoding the chemokine CXCL9 and an anti-PD-1 Single-Chain Variable Fragment (scFv) has shown the ability to significantly remodel the tumor immune microenvironment, promoting T cell infiltration and sustained anti-tumor activity [100].

Another promising platform involves alphavirus-derived replicon particles (VRPs) encoding HER2 [98]. VRPs efficiently stimulate innate immunity and antigen expression without genomic integration. A phase II study (NCT03632941), with pending results, is evaluating the VRP-HER2 vaccine with pembrolizumab, aiming to leverage viral vector–mediated immunogenicity alongside checkpoint blockade.

b) New Adjuvants

To overcome poor antigen presentation and immunosuppressive TME efforts should focus on optimizing combinations of tumor antigens and potent immunologic adjuvants. This includes the development of novel adjuvants specifically designed to target a broad range of subunit antigens, peptides, and proteins, thereby improving antigen uptake and activation of APCs. In addition, booster vaccinations have shown potential in sustaining immune activation and reducing recurrence rates. Therefore, selecting the most effective adjuvant strategies—both in initial dosing and in booster regimens—is essential to maximizing long-term immune protection and therapeutic efficacy [101, 102].

Traditional adjuvants, such as the water-in-oil emulsions Montanide ISA 720 and Montanide ISA-51, function by forming a depot at the injection site [103]. This depot effect traps soluble antigens, slows their drainage to local lymph nodes, and induces localized inflammation, facilitating gradual antigen release and prolonged immune stimulation [103]. However a new generation of adjuvants has emerged. These include toll-like receptor (TLR) agonists—such as monophosphoryl lipid A (MPLA)—which mimic microbial signals to activate DCs and promote antigen presentation [95]. MPLA is already incorporated into approved vaccines due to its potent immunostimulatory properties and favorable safety profile. Other promising adjuvant classes include Stimulator of Interferon Genes (STING) agonists, which trigger type I interferon production; saponins like QS-21, known to enhance cytotoxic T cell responses [96]; pathogen-associated molecular patterns (PAMPs) [34]; and CD40 agonists [104], which directly activate antigen-presenting cells. These innovative adjuvants collectively have the potential to significantly amplify both humoral and cellular immune responses. Lastly, several cytokines have demonstrated potent adjuvant properties and are currently under investigation such as interleukin-2 (IL-2) [105], interferons (IFNs) [106] and interleukin-12 (IL-12) [107]. These cytokines enhance vaccine efficacy by recruiting DCs to the injection site, promoting their maturation, and facilitating antigen presentation—thereby strengthening the adaptive immune response. However, some cytokines also exert complex and contradictory immunomodulatory effects. For example, IL-2 and GM-CSF can contribute to the expansion of MDSCs, which are known to dampen T cell–mediated antitumor responses and promote immune evasion [108]. These dual roles highlight the need for careful dosing and combination strategies to harness cytokine adjuvants effectively while minimizing immunosuppressive side effects. Combining peptide vaccines with these potent adjuvants and other immunomodulatory agents could significantly improve their clinical performance and therapeutic potential in BC treatment [109].

c) Next-Generation Delivery Systems

Advances in chemical and synthetic material technologies will play a critical role in improving the targeted delivery of these antigens to immune cells. Conjugating antigenic peptides with cell-penetrating peptides (CPPs) has emerged as a promising strategy to enhance peptide vaccine delivery [110]. The combination of antigenic epitopes with CPPs, such as the S4 transduction domain, has shown the ability to improve peptide loading and T cell activation, enhancing the immunogenicity of vaccines [111].

Furthermore, biomimetic delivery systems are being developed to overcome anatomical obstacles such as the blood–brain barrier (BBB). A notable example includes neutrophil membrane-coated circRNA nanoparticles (NCNPs) engineered with anti-HER2 scFv. These 'cloaked' nanoparticles exploit the natural tumor-homing capabilities of neutrophils to deliver immunotherapeutic circRNA directly to brain metastatic sites, addressing a major unmet clinical need in HER2 + disease [100, 112].

d) Neoantigen Discovery and Epitope Optimization

Neoantigens offer precise targeting and minimal off-target effects. Advances in sequencing and computational epitope prediction enable personalized vaccines that account for tumor heterogeneity and clonal evolution [113]. Optimizing both MHC class I and II–restricted epitopes may enhance T cell diversity and durability of responses [114]. Emerging preclinical data have identified the oncogenic isoform HER2Δ16. HER2Δ16 not only drives oncogenic signaling but also confers resistance to standard HER2-directed treatments [115]. Vaccines directed against HER2Δ16 have demonstrated the capacity to elicit potent antitumor immune responses, in part by generating TILs specifically reactive to this isoform [115].

e) New Combinations

CVs efficacy may be significantly enhanced when combined with other immunotherapies. These combinations can address multiple mechanisms of immune resistance simultaneously, potentially leading to more durable and effective antitumor responses. Importantly, combining HER2Δ16-targeted vaccination with immune checkpoint blockade—such as anti–PD-1 therapy—has been shown to enhance long-term tumor control [116].

Among emerging combinatorial strategies, the integration of CVs with ICIs represents a particularly compelling approach supported by a strong biological rationale. Immune checkpoint blockade is most effective in the presence of pre-existing tumor-specific T cell responses and an inflamed tumor microenvironment. In this context, cancer vaccines enhance antigen presentation and promote the priming of tumor-specific T cells, thereby increasing T cell infiltration into otherwise poorly immunogenic tumors. However, vaccine-induced immune responses are often insufficient to achieve durable clinical benefit due to the immunosuppressive TME, which limits T cell functionality. ICIs can overcome these limitations by restoring T cell activity and reversing inhibitory signaling pathways such as PD-1/PD-L1, thereby enhancing the effector phase of the antitumor immune response.

This combination is therefore mechanistically synergistic, as vaccines enhance immune priming while ICIs sustain and reinvigorate T cell function, potentially converting “cold” tumors into “hot” tumors. Early clinical evidence suggests that treatment sequencing may influence efficacy, supporting the design of rational combination trials incorporating vaccines and ICIs with appropriate patient selection and biomarker strategies [117].

Moreover, a recent study investigated HER2-specific vaccination to prime T cells for ex vivo expansion in patients with advanced HER2 + BC. In this approach, HER2-targeted vaccination was combined with interleukin-2 (IL-2) and interleukin-12 (IL-12) to expand autologous HER2-specific T cells ex vivo. The strategy successfully primed T cells for expansion, promoted epitope spreading, and enhanced the persistence of the infused T cells post-transfer. These results highlight the importance of epitope spreading—a phenomenon where immune responses broaden beyond the initial targeted epitope—in strengthening overall antitumor immunity and support the potential of combining vaccine-primed T cell therapies with other immune-based modalities to improve clinical outcomes in HER2 + MBC [118].

However, the success of these combinations also depends on unravelling the circRNA-mediated resistance mechanisms that span the HER2 spectrum. For instance, the engineering of circRNA-loaded nanoparticles (NCNPs) for the targeted delivery of CXCL9 and anti-PD-1 to circumvent the BBB in brain metastases represents an innovative delivery system that is, in itself, a potent combination therapy. Conversely, recent evidence shows that circ-*β*-TrCP can drive resistance to trastuzumab by encoding a novel protein isoform (*β*-TrCP-343aa) that stabilizes the antioxidant regulator NRF2 [119].

Similarly, in HER2-low settings, the circular RNA crVDAC3 has been found to mediate resistance to Trastuzumab Deruxtecan (T-DXd) by inhibiting HSPB1-dependent ferroptosis. Integrating vaccine therapies with small molecules or inhibitors specifically targeting these circRNA-protein axes-such, as the use of Paritaprevir to restore ferroptosis, could represent a crucial strategy to re-sensitize tumors and maximize the synergistic potential of vaccine-induced anti-tumor immunity [102].

In parallel, advancing strategies to reinvigorate tumor-specific T cells that have entered states of exhaustion or senescence will be crucial [120]. Reversing these dysfunctional phenotypes may enhance the formation of long-lasting memory T cells, which are essential for durable immune surveillance and sustained tumor control.

**Fig. 3:**
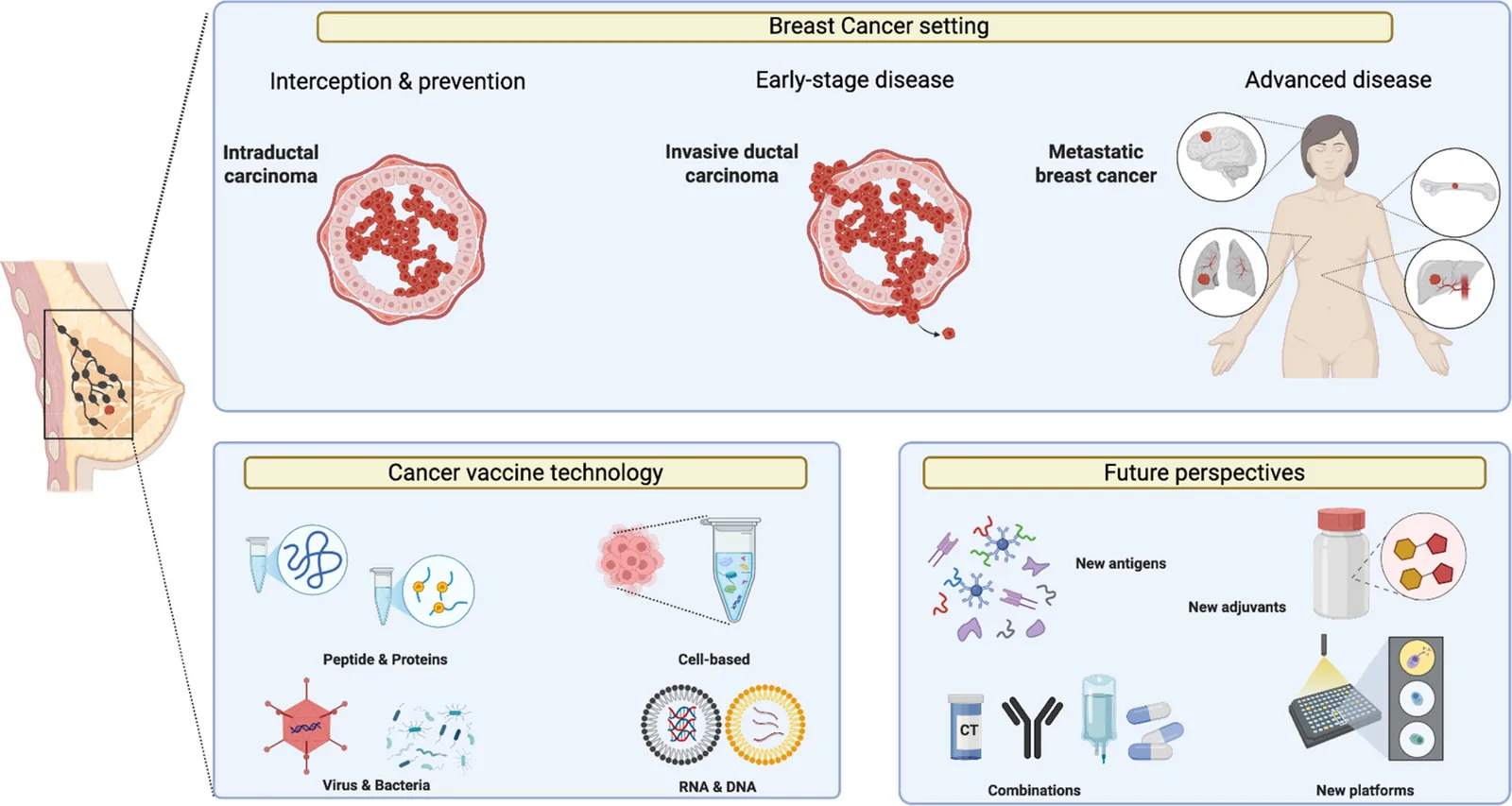
The landscape of cancer vaccine strategies in BC. Overview of cancer vaccine strategies across the breast cancer disease continuum. The figure illustrates the potential application of cancer vaccines from interception and prevention to early-stage and advanced breast cancer, highlighting established and emerging vaccine technologies, including peptide/protein-based, cell-based, viral/bacterial vector, and RNA/DNA platforms, as well as future directions such as novel antigens, adjuvants, combination approaches, and next-generation delivery systems

Figure 3 provides a comprehensive overview of how current technological advancements and future clinical perspectives are converging to redefine the treatment landscape of breast cancer.

## Conclusions

Despite ongoing challenges, the promise of harnessing the immune system against BC remains strong. Research is increasingly focused on improving vaccine efficacy, especially in HER2⁺ and TNBC subtypes where immune modulation may be most impactful. Advances in delivery systems, novel adjuvants, and personalized neoantigen approaches are expanding the potential to induce durable antitumor responses, even in immunologically “cold” tumors.

Translation into practice will require validation trials but growing preclinical and early-phase evidence supports continued investment in vaccine strategies. Integrating CVs into multimodal regimens—alongside targeted agents, checkpoint inhibitors, or adoptive cell therapies—may be key to maximizing benefit.

In summary, cancer vaccinology offers a promising avenue to improve long-term survival and quality of life for breast cancer patients, with continued innovation and clinical testing critical to realizing this potential.

## Data Availability

No datasets were generated or analysed during the current study.
